# Tethering Complexes in the Arabidopsis Endomembrane System

**DOI:** 10.3389/fcell.2016.00046

**Published:** 2016-05-19

**Authors:** Nemanja Vukašinović, Viktor Žárský

**Affiliations:** Laboratory of Cell Morphogenesis, Department of Experimental Plant Biology, Faculty of Science, Charles UniversityPrague, Czech Republic

**Keywords:** Tethering complexes, TRAPP, Dsl1, Exocyst, COG, GARP, HOPS, CORVET

## Abstract

Targeting of endomembrane transport containers is of the utmost importance for proper land plant growth and development. Given the immobility of plant cells, localized membrane vesicle secretion and recycling are amongst the main processes guiding proper cell, tissue and whole plant morphogenesis. Cell wall biogenesis and modification are dependent on vectorial membrane traffic, not only during normal development, but also in stress responses and in plant defense against pathogens and/or symbiosis. It is surprising how little we know about these processes in plants, from small GTPase regulation to the tethering complexes that act as their effectors. Tethering factors are single proteins or protein complexes mediating first contact between the target membrane and arriving membrane vesicles. In this review we focus on the tethering complexes of the best-studied plant model—*Arabidopsis thaliana*. Genome-based predictions indicate the presence of all major tethering complexes in plants that are known from a hypothetical last eukaryotic common ancestor (LECA). The evolutionary multiplication of paralogs of plant tethering complex subunits has produced the massively expanded EXO70 family, indicating a subfunctionalization of the terminal exocytosis machinery in land plants. Interpretation of loss of function (LOF) mutant phenotypes has to consider that related, yet clearly functionally-specific complexes often share some common core subunits. It is therefore impossible to conclude with clarity which version of the complex is responsible for the phenotypic deviations observed. Experimental interest in the analysis of plant tethering complexes is growing and we hope to contribute with this review by attracting even more attention to this fascinating field of plant cell biology.

## Introduction−a common set of LECA tethering complexes and their GTPase regulators in plants

Plant cells are encased in cell walls, and regulation of plant growth and development thus relies upon oriented cell division and cell expansion without the contribution of cellular movements that are typical for animals. To achieve this precisely positioned cell division and cell expansion, endomembrane compartment identity and dynamics are controlled by the intricate regulatory networks, involving not only cytoskeletal and membrane associated proteins, but also membrane lipids. Major progress in the analysis and understanding of these processes was achieved using yeast and animal models, and in the case of plant cells most studies have used the Arabidopsis model. In keeping a distinct compartmental identity within the constant flow of membrane exchange between specific endomembrane compartments in the cell, Ras-related in brain (RAB) small GTPases, along with ADP-ribosylation factors (ARF) and Ras-like GTP binding protein (RHO) GTPases, seem to play central roles as local membrane identity organizers (Zerial and McBride, 2001; for a review of RAB GTPases functional cycle regulation and effectors biology in plants see Woollard and Moore, 2008).

The logistics of inter-compartmental membrane transport require well-defined specific mechanisms for recognition between the membrane transport containers and the proper target endomembrane. Here is the major realm of tethering factors function within the cell: they mediate, either as single long rod-like proteins or protein complexes, the first specific contact between the arriving membrane vesicle and the target membrane. This is often integrated into the functional cycle of specific RAB GTPases with tethering complexes functioning as guanine nucleotide exchange factors (GEFs) for RABs or as effectors of activated RABs specific for each transport step between endomembrane compartments (Grosshans et al., 2006).

Sets of endomembrane compartments regulators traceable to last eukaryotic common ancestor (LECA) are now known to include all basal ARF, RAB, and RHO GTPases classes, as well as their regulators and effectors, including tethering factors. Tethering complexes function depending on the contexts both as small GTPase regulators (e.g., GEFs) and effectors (see above Koumandou et al., 2007; Markgraf et al., 2007; Eliáš, 2010; Eliáš et al., 2012; Klinger et al., 2013). In fact, a trend of lineage specific losses and intra-subclass diversifications seems to be a dominant feature in the evolution of small GTPases (Eliáš, 2010). As tethering factors are generally both effectors and regulators of small GTPases in the RAB and RHO families, it is interesting to see the status of these membrane-recruitable molecular switches in plants.

There are 12 ARF GTPases (as well as several ARF-like proteins—ARLs) encoded in the *Arabidopsis thaliana* genome and their comparative phylogenetic analysis along with specificities in ARF GEF function (e.g., Teh and Moore, 2007) indicate that processes regulated by this class of proteins have undergone plant-specific evolutionary diversification (for a recent review see Yorimitsu et al., 2014).

In plants, the RAC/RHO common ancestor diversified into the plant-specific version of RHO GTPases called “RHO of plants”—ROP GTPases (Zheng and Yang, 2000). Two major angiosperm ROP classes are clearly recognized—type-I already present in the early diverging basal land plant lineages (mosses, lycophytes), and higher plant-specific type-II, obviously an evolutionary outcome of the duplication and functional diversification of type-I predecessors somewhere at the post-lycophytes phylogenetic stage (Fowler, 2010). While class I ROPs are “classical” C-terminal cystein-prenylated proteins, class II ROPs are attached to the endomembranes by S-acylation of hypervariable domain GC-CG box Cys residues (Ivanchenko et al., 2000; Lavy et al., 2002; Sorek et al., 2011; Yalovsky, 2015). Grasses are dominated by type-II ROP GTPases, but most of the dicotyledonous plants are dominated by type-I ROP GTPases, indicating that there is possibly no deep split of functional tasks between type-I and type-II ROPs (Fowler, 2010).

Interestingly, a prenylation-independent type of membrane localization did also evolve among plant RAB GTPases. One of three Arabidopsis RAB5 homologs called ARA6, requires N−terminal acylation and thus represents a plant-specific mechanism of RAB functional cycle regulation between endosomes and plasma membrane (PM) (the “conventional” prenylated RAB5 functions between endosomes and the vacuole in Arabidopsis; Ueda et al., 2001). The plant RAB subclasses underwent specific evolutionary histories (for the recent overview see Petrželková and Eliáš, 2014). Most importantly, very early duplication of RAB11 (RAB-A) subclass resulted in three basal RAB11 paralogs in early land plants followed by further duplications (and possibly subfunctionalizations) resulting in as many as 26 RAB11/RAB-A paralogs encoded in the *A. thaliana* genome (Rutherford and Moore, 2002; Petrželková and Eliáš, 2014). Mere evolutionary amplification of most probably exocytotic/PM recycling RAB11 paralogs implies the importance of cortical domain-specific membrane targeting and recycling for plant cell morphogenesis, function and survival (Zárský et al., 2009).

**Figure 1:**
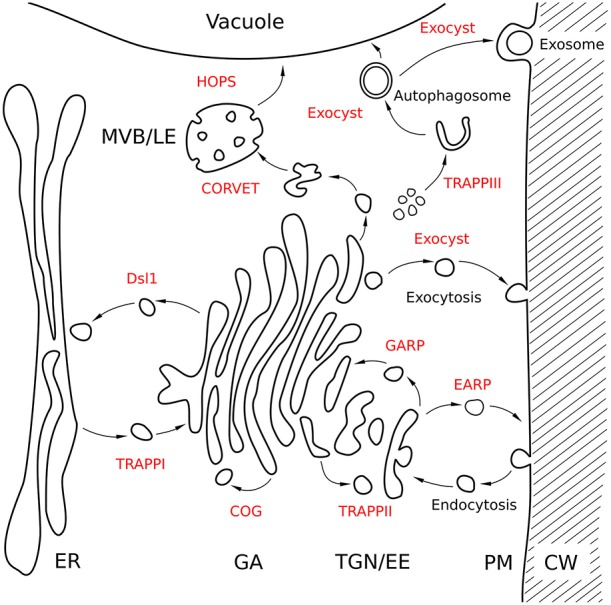
**Putative locations and functions of tethering complexes in a schematic Arabidopsis cell**. The endomembrane system of a plant cell is shown with the positions of tethering complexes in specific transport steps. Arrows indicate putative directions of action of tethering complexes. For some complexes no experimental data are available and assumptions are completely based on yeast and animal data. CW, cell wall; ER, endoplasmic reticulum; GA, Golgi apparatus; MVB/LE, multivesicular body/late endosome; PM, plasma membrane; TGN/EE, trans-Golgi network/early endosome.

Early in eukaryogenesis, a set of multisubunit tethering complexes was established in the LECA and evolved in lineage-specific ways, often including evolutionary losses of some complexes, esp. in specialized parasitic protists (Koumandou et al., 2007; Markgraf et al., 2007; Klinger et al., 2013). Several complexes seem to be evolutionary related and are currently called “CATCHR” complexes (complexes associated with tethering containing helical rods). They usually comprise of either four—Golgi-associated retrograde protein (GARP) and endosome-associated retrograde protein (EARP) complexes or eight subunits—conserved oligomeric Golgi (COG) complex and exocyst (for their position within the cell see Figure 1). The three-subunit, depends on SLY1-20 (Dsl1) complex is also categorized within this group. These complexes function in the secretory/biosynthetic pathway (Figure 1). The class C core vacuole/endosome tethering (CORVET) and homotypic fusion and protein sorting (HOPS) from the second group of multisubunit tethering complexes are required for endosome-vacuolar/lysosomal transport (Figure 1), and the consecutively assembling trafficking protein particle (TRAPP) I–III complexes form another group also involved in biosynthetic pathways as well as autophagy pathways (Bröcker et al., 2010). All these complexes are conserved in plant genomes (Koumandou et al., 2007; Klinger et al., 2013). However, with a few exceptions, the understanding of the regulation of plant tethering complexes, including the input of small GTPases, is almost non-existent.

The aim of our review is therefore not only to summarize relevant current knowledge, in particular all published experimental observations on tethering complexes in plants (which, in fact, means mostly in Arabidopsis), but also to encourage new interest in the exploration of this field of plant cell biology. We refer to published data, without checking published genomic predictions for their accuracy or completeness, and only in a few specific cases have we searched for new complex subunits in the *A. thaliana* genome using BLAST. Consequently we cannot exclude that some new relevant proteins are still awaiting discovery. However, good support for the physical existence for all LECA tethering complexes (and for some GTPases interactions) is documented by large scale interactome analyses (e.g., BIOGRID—http://thebiogrid.org/).

Without the mechanistic understanding of the endomembrane vesicle targeting and tethering in plant cells, many agronomically important features including biomass accumulation, stress and defense reactions will not be properly amenable to rational breeding or modification.

## TRAPP complexes

Three forms of TRAPP complex exist in yeast: TRAPPI that functions in endoplasmic reticulum (ER) to Golgi apparatus (GA) transport, TRAPPII which operates in trans-Golgi trafficking and TRAPPIII which is involved in autophagosome formation (Sacher et al., 2008; Lynch-Day et al., 2010). TRAPPI was shown to work as a GEF for the Ypt1p GTPase that regulates an ER-to-GA transport step, while TRAPPII is a GEF for the Ypt31/32p GTPase functional pair that regulates trans-Golgi trafficking (Jones et al., 2000). Bet3, Bet5, Trs20, Trs23, Trs31, and Trs33 subunits are shared by all three complexes, Trs65 (present only in yeast), Trs120, and Trs130 are specific to TRAPPII, while Trs85 specifies TRAPPIII complex (Sacher et al., 2008; Lynch-Day et al., 2010).

## TRAPPI

In yeast, TRAPPI is involved in the ER-to-GA transport by mediating the tethering of coat protein complex II (COPII)-coated vesicles at the *cis*-Golgi domain, via interaction of Bet3p subunit with the COPII coat adaptor subunit Sec23p (Cai et al., 2007). In addition, TRAPPI functions as a GEF for the Rab GTPase Ypt1p. Bet3p, Bet5p, and Trs23p subunits of the complex directly interact with Ypt1p to facilitate guanine nucleotide exchange—i.e., Ypt1p activation (Cai et al., 2008). Predicted in the *A. thaliana* genome and recovered in large scale interactome databases, all six subunits of the TRAPPI complex possibly exist and function between the ER and GA in land plants (Koumandou et al., 2007; Thellmann et al., 2010; Klinger et al., 2013; Table 1). The subunit TRS85, considered originally as a TRAPPI subunit, was shown to be a specific subunit of the TRAPPIII version of the complex (Lynch-Day et al., 2010; see below). Arabidopsis LOF mutants defective in the BET3, BET5, TRS31, or TRS33 subunits all displayed weak deviations from the normal course of cytokinesis. TRS33 LOF mutant displayed relatively higher penetrance of the cytokinetic defect as compared to others, with cell wall stubs and “floating walls” (i.e., initiated but unfinished cell plates) observed (Thellmann et al., 2010). Cytokinetic defects in TRAPPII mutants were generally more pronounced (Thellmann et al., 2010; see below). TRAPPI is acting as a GEF for RAB1 GTPases in yeast and animals and RAB1/RAB-D orthologs are well conserved in plants (Woollard and Moore, 2008). Therefore, it is possible that this GEF activity is also conserved in plants.

**Table 1:** **TRAPPI, TRAPPII, and TRAPPIII subunits from yeast and human and their possible homologs in ***Arabidopsis thaliana*****.

	**Yeast/Human subunit**	**Plant subunit**	**Potential *A. thaliana* homolog**
TRAPPI	Bet5/TRAPPC1	BET5	At1g51160
	Trs20/TRAPPC2	TRS20	At2g20930
	Bet3/TRAPPC3	BET3	At5g54750
	Trs23/TRAPPC4	TRS23	At5g02280
	Trs31/TRAPPC5	TRS31	At5g58030
	Trs33/TRAPPC6	TRS33	At3g05000
	-/TRAPPC11	NA	At5g65950
	-/TRAPPC12	NA	At4g39820
TRAPPII	Trs65/-	–	–
	Trs120/TRAPPC9	TRS120/VAN4	At5g11040
	Trs130/TRAPPC10	TRS130/CLUB	At5g54440
	TCA17/TRAPPC2L	TRS20	At2g20930
TRAPPIII	Trs85/TRAPPC8	TRS85	At5g16280

## TRAPPII

In yeast cells the TRAPPII complex acts as a GEF for Rab GTPase Ypt31p (RAB11 related) and mediates intra- and post-Golgi trafficking (Yu and Liang, 2012). TRAPPII-specific subunits Trs120p and Trs130p are required for Ypt31p GEF activity while at the same time they are necessary for inhibiting Ypt1 GEF activity of this version of the TRAPP complex (Morozova et al., 2006). In Arabidopsis, the TRAPPII complex regulates intra-GA membrane transport (with the contribution to GA exit and autophagy; Yu and Liang, 2012) and is composed, as in other eukaryotes, of all TRAPPI subunits with additional specific subunits. However, in contrast to three known TRAPPII specific-subunits in yeast, only two are encoded by the *A. thaliana* genome—the TRS65 homolog is missing (Thellmann et al., 2010; Klinger et al., 2013). TRS65 is present only in fungi, and it was recently proposed that the newly discovered TRAPPII subunit TCA17 is the major factor of TRAPPII assembly and it is also predicted to function in plants (Klinger et al., 2013; see Table 1). TRAPPI and TRAPPII subunits were isolated in a proteomic characterization of the TGN/SYP61 compartment in Arabidopsis, indicating that in plants, like in other organisms, TRAPPII might also be involved in post-Golgi membrane traffic (Drakakaki et al., 2012). Arabidopsis LOF mutants *trs120* and *trs130* (allelic to *club2*—Söllner et al., 2002; Jaber et al., 2010) display very strong cytokinesis defects resulting in seedling lethality (Thellmann et al., 2010; Qi et al., 2011). Focusing on several independent *TRS120* T-DNA insertional alleles, Thellmann et al. (2010) frequently found cell wall stubs and incomplete cross walls. Phragmoplast vesicles accumulated at the division plane during cell division, but did not assemble a normal cell plate, indicating that TRS120 is required for cell plate biogenesis. Weaker transmission defects of TRAPPII subunits LOF mutants were also observed, indicating negative effects on the gametophytic development and sexual process (Thellmann et al., 2010). In TRAPPII mutants specifically post-GA vesicle trafficking to the plasma membrane (PM) is affected, while vacuolar membrane delivery seems to be intact (Qi et al., 2011).

Naramoto et al. (2014) implicated TRS120 in xylem differentiation, and a detailed comparative study of the dynamics of TRAPPII and exocyst complex subunits (see below) indicated intimate collaboration of these two tethering complexes in Arabidopsis cytokinesis; including direct interaction involving specific EXO70 and TRS120 subunits (Rybak et al., 2014). The ability of TRS120 and TRS130 subunits to pull-down the exocyst complex is also well documented in the BIOGRID database. Both complexes are prominently localized to the initiating cell plate; during phragmoplast extension, TRAPPII prevails at the cell plate over the exocyst; which dominates final insertion and maturation phases of new cell wall ontogenesis (Rybak et al., 2014). In contrast to plants, a recent study on roles of TRAPPII and exocyst during cytokinesis in fission yeast *S. pombe* does not support their direct physical interaction (Wang et al., 2016). However, the study confirms involvement of both TRAPPII and exocyst in cytokinesis and further research in plants and other eukaryotes will be needed to determine the details of this cooperation. Comparing trafficking of auxin efflux carriers PIN FORMED (PIN)1 and PIN2, or influx carriers AUX1 or AUX2, it was reported that only AUX2 and PIN2 PM delivery is TRAPPII dependent (Qi et al., 2011; Qi and Zheng, 2011). Colocalization and possibly interaction of trans-Golgi network (TGN)-derived RAB-A1c vesicles and TRS130 was also observed (Qi et al., 2011). Moreover, the constitutively activated (CA) version of RAB-A1c GTPase partially rescued phenotypes associated with Arabidopsis TRS130 LOF, providing support for the expected upstream function of TRAPPII complex in the activation of this GTPase (Qi et al., 2011). Specificity toward RAB-A1c (i.e., RAB11 or YPT31/32 homologs) was confirmed by the comparison with RAB-D (a plant RAB1 homolog) showing no interaction (Qi and Zheng, 2011). Detailed localization studies of RAB-A1c and other closely related RAB-A1 GTPases indicates its maximal presence on mobile structures adjacent to the TGN highlighted by the vesicle—Soluble N-Ethylmaleimide-Sensitive Factor Attachment Protein Receptor (v-SNARE) markers VAMP724 and VAMP722, and are possibly exocytosis-related containers (Asaoka et al., 2013). A putative TRAPPC11 subunit known from the animals (Yu and Liang, 2012), currently unknown in plants, might be encoded by At5g65950 in Arabidopsis—coexpression data indicate possible correlation with secretory genes including TRS120.

## TRAPPIII

The TRAPPIII complex consists of all TRAPPI subunits as a core with one additional specific subunit, TRS85, originally supposed to be part of the TRAPPI complex (Yu and Liang, 2012; Klinger et al., 2013). Analyses in yeast and mammals recently indicated that the dominant function of the TRAPPIII complex is in autophagy-related membrane transport and possibly also exocytotic GA export (Yu and Liang, 2012; for. rev. see Klinger et al., 2013). The only predicted ortholog from the Arabidopsis genome (see Table 1—At5g16280) was surprisingly recovered, along with other TRAPP subunits, in the proteomic analysis of the purified SYP61 positive TGN compartment (Drakakaki et al., 2012). In a recent proteomic study of Arabidopsis endosomal and secretory pathways, the TRS85 subunit was found in the RAB-D2a/ARA5-labeled affinity purified membrane fraction (Heard et al., 2015). RAB-D2a/ARA5 GTPase is a marker for the TGN/EE compartment, further supporting the idea that TRS85 might have a different role in plants besides its involvement in autophagy. The RAB1 GTPase (Arabidopsis homologs are also called RAB-D, see above) is directly activated by TRAPPIII GEF activity to localize at the forming phagophore (Lynch-Day et al., 2010). A putative ortholog of the N′-terminal half of the mammalian TRAPP complex subunit TRAPPC12 is proposed to be involved in autophagy (Yu and Liang, 2012) and might be encoded by the At4g39820 locus in Arabidopsis (Table 1).

## Dsl1 complex

The Dsl1 complex consists of three subunits in yeast (Dsl1p, Tip20p, and Dsl3/Sec39) and mammals (ZW10, RINT-1 and NAG), and it operates in the retrograde transport between the GA and ER (Hirose et al., 2004; Kraynack et al., 2005; Aoki et al., 2009). Dsl1 captures vesicles via its interaction with the coat protein complex I (COPI) coat (Zink et al., 2009) and interacts with ER SNAREs (Kraynack et al., 2005). It is proposed that through these interactions the Dsl1 complex coordinates tethering, uncoating, SNARE assembly, and membrane fusion (Ren et al., 2009).

The Dsl1 complex, along with the exocyst, is an exception in respect to plant-related data available as insights into its composition and function in Arabidopsis have been reported (Tagaya et al., 2014). The first Dsl1 complex subunit related to the yeast TIP20 subunit was characterized as *maigo2* (*mag2*) mutant within a screen for mutants affected in the transport and deposition of seed storage proteins precursors 2S albumin and 12S globulin in Arabidopsis (Li et al., 2006). Three known subunits of the Dsl1 complex are present in the plant complex and were recovered as TAP-tagged MAIGO2 interacting proteins/MIPs (Li et al., 2013). Surprisingly an additional fourth subunit—MIP3, with Sec1/Munc18 domain, not known from other model eukaryotes, is part of the complex in Arabidopsis (Li et al., 2013; see Table 2). MAG2 is localized partially at the ER periphery and can interact with ER t-SNAREs AtSEC20 and SYP81/AtUFE1 (Li et al., 2006). The MIP1 subunit, ortholog of Zeste–White 10 (ZW10) subunit of the mammalian complex, was shown to co-localize with the ER as well. The MIP2 subunit of the MAG2 complex harbors a Sec39 domain as expected for Dsl3 (Li et al., 2013). In conclusion, there is an obvious Arabidopsis homolog of the yeast and mammalian Tip20p–Dsl1p–Sec39p/RINT1–ZW10–NAG complex with a new fourth MIP3 subunit featuring a Sec1/Munc18 domain (Li et al., 2013). Comparison of LOF mutants in these subunits indicates that all of them are involved in seed storage protein maturation, as all mutants developed abnormal ER-derived storage protein body structures (MAG bodies) in seeds (Li et al., 2013). Certainly, the function of the Dsl1 complex in plants is more general and analysis of *mag2* and other subunit mutants (including partially redundant *mag2l*–*mag2* related Arabidopsi*s* paralog) indicates that it is also involved in stress, abscisic acid and gibberellin dependent signaling (Zhao Y. et al., 2013; Zhao and Lu, 2014). All these mutants are not only more sensitive to ER stress, but seem to display a constitutive ER-stress response (Zhao Y. et al., 2013). This might indicate a putative role of the Dsl1 complex also in the plant autophagy pathway (Tagaya et al., 2014). However, general ER network structure and vacuolar or secretory cargos delivery seem to remain unaffected in Dsl1 LOF Arabidopsis mutants (Zhao Y. et al., 2013).

**Table 2:** **Dsl1 subunits from yeast and human and their possible homologs in ***Arabidopsis thaliana*****.

**Yeast/Human subunit**	**Plant subunit**	**Potential *A. thaliana* homolog**
Tip20/RINT1	MAG2	At3g47700
Dsl1/ZW10	MIP1	At2g32900
Sec39/NAG	MIP2	At5g24350
–	MIP3	At2g42700

## COG complex

The COG complex mediates retrograde vesicular trafficking at the level of the GA (Ungar et al., 2006). In yeast and mammals, this tethering complex is composed of eight subunits that form two lobes—lobe A (COG1–4) and B (COG5–8) (Ungar et al., 2002; Fotso et al., 2005). It coordinates vesicle tethering within the GA through physical interactions with various endomembrane trafficking regulators such as SNAREs, Rab GTPases, COPI and golgins (Laufman et al., 2009; Miller et al., 2013). Mutations in both lobes lead to glycosylation defects in COG mutant cells, which are caused by the aberrant recycling of glycosylation enzymes between GA cisternae (Pokrovskaya et al., 2011). All eight COG subunits are fully retained in most of the eukaryotes (Koumandou et al., 2007), including plants, where amino acid identity between putative subunits of *A. thaliana* and their human counterparts ranges between 20 and 34% (Latijnhouwers et al., 2005; see Table 3). Based on publicly available data, AtCOG6 physically interacts with AtCOG2 and AtCOG8 (http://thebiogrid.org/), indicating the existence of a functional COG complex in plants. Nevertheless, only a few studies have focused on the role of the COG complex in membrane trafficking events in plant cells. Ishikawa et al. (2008) identified a mutation in the Arabidopsis *embryo yellow, EYE* gene, which encodes a putative AtCOG7 subunit. *eye* mutant plants are bushy, have aberrant organization of shoot apical meristem and altered cell wall composition. Also, the ERD2-GFP marker which is targeted to both GA and ER in Arabidopsis cells (Boevink et al., 1998) is mislocalized in *eye* mutants to the ER only, implying that EYE is necessary for maintenance of normal GA structure. In mammalian cells the N-terminus of COG7 interacts with COG5, and disruption of this interface leads to the aberrant cell surface glycosylation (Ha et al., 2014). Interestingly, overexpression of the conserved N-terminus of AtCOG7 homolog in Arabidopsis (residues 1–123) turned out to be sufficient for the complementation of the *eye* mutant phenotype. Several studies indicate that the plant COG complex gained additional functions, along with its involvement in retrograde GA trafficking. HvCOG3 is essential for resistance to fungal penetration into the host cell of barley (Ostertag et al., 2013) and AtCOG2 was attributed the role in the recruitment of the exocyst complex to the sites of secondary cell wall deposition in Arabidopsis xylem cells (Oda et al., 2015; see below). This finding is surprising, as the role of the COG complex in plants seems to be in a conflict with the canonical role of this complex in retrograde trafficking. However, Miller et al. (2013) showed that mammalian Rab4a, which is involved in recycling from endocytic compartments to the plasma membrane (Hutagalung and Novick, 2011), interacts with the COG6 subunit, indicating that the COG complex might also be involved in anterograde secretory events in mammalian cells as an effector of RAB4a. RABF-2b/ARA7 positive compartments seem to act as a hub for COG-exocyst cross-talk in Arabidopsis, since the RABF-2b/ARA7 compartment affinity purification leads to enrichment of both COG and exocyst components (Heard et al., 2015). GARP complex components are also abundant at the same compartment (Heard et al., 2015) and it would be interesting to see if the GARP complex is a part of this putative cooperation network of tethering complexes. These and some other instances of inter-complex cross-talk indicate a possible intricate network of tethering complex co-regulation.

**Table 3:** **COG subunits from yeast and human and their possible homologs in ***Arabidopsis thaliana*****.

**Yeast/Human subunit**	**Plant subunit**	**Potential *A. thaliana* homolog**
Cog1/COG1	NA	At5g16300
Cog2/COG2	COG2	At4g24840
Cog3/COG3	NA	At1g73430
Cog4/COG4	NA	At4g01400
Cog5/COG5	NA	At1g67930
Cog6/COG6	NA	At1g31780
Cog7/COG7	COG7/EYE	At5g51430
Cog8/COG8	NA	At5g11980

## The exocyst complex

The exocyst is an octameric complex mediating tethering of secretory vesicles to the PM, involved intimately also in the regulation of cell polarity (He and Guo, 2009; Heider and Munson, 2012). Its eight subunits (Sec3, Sec5, Sec6, Sec8, Sec10, Exo70, and Exo84) form a stable complex in yeast (Heider et al., 2016). Yeast and mammalian Sec3 and Exo70 subunits interact directly with the activated RHO GTPases and membrane lipids and are proposed to serve as landmarks for delivery of secretory vesicles to a specific PM domain (Boyd et al., 2004; Wu et al., 2008; Pleskot et al., 2015) Rab GTPases Sec4 (in yeast) or RAB11 (in animals) and v-SNARE Snc2 interact with Sec15 and Sec6 respectively, to link the complex to the secretory vesicle (Guo et al., 1999; Shen et al., 2013).

Due to its engagement in the final stages of exocytosis, i.e., also in the cell wall biogenesis, plant cell cytokinesis and defense, the plant exocyst complex has attracted more attention as compared to other tethering complexes in plants. The first Arabidopsis genome analysis (Eliáš et al., 2003) immediately indicated a peculiar feature of the land plant exocyst complex - a multiplicity of EXO70 paralogs with more than twenty-three genes in Arabidopsis and forty genes encoded by the rice genome (Cvrčková et al., 2012; Table 4). The first phenotypic analyses of LOF exocyst subunit mutants in Arabidopsis and maize clearly supported the expected involvement of the exocyst in the targeting of secretory vesicles that regulate plant cell polarity (Cole et al., 2005; Wen et al., 2005; Synek et al., 2006). The plant exocyst was partially purified and shown to exist as a biochemical entity with all eight basic subunits present (Hála et al., 2008; Fendrych et al., 2010). Over the last ten years its participation has been documented in many exocytosis-related processes in plants (reviewed in Zárský et al., 2009, 2013).

**Table 4:** **Exocyst subunits from yeast and human and their possible homologs in ***Arabidopsis thaliana*****.

**Yeast/Human subunit**	**Plant subunit**	**Potential *A. thaliana* homolog**
Exo70/EXO70	EXO70A1	At5g03540
-II-	EXO70A2	At5g52340
-II-	EXO70A3	At5g52350
-II-	EXO70B1	At5g58430
-II-	EXO70B2	At1g07000
-II-	EXO70C1	At5g13150
-II-	EXO70C2	At5g13990
-II-	EXO70D1	At1g72470
-II-	EXO70D2	At1g54090
-II-	EXO70D3	At3g14090
-II-	EXO70E1	At3g29400
-II-	EXO70E2	At5g61010
-II-	EXO70F1	At5g50380
-II-	EXO70G1	At4g31540
-II-	EXO70G2	At1g51640
-II-	EXO70H1	At3g55150
-II-	EXO70H2	At2g39380
-II-	EXO70H3	At3g09530
-II-	EXO70H4	At3g09520
-II-	EXO70H5	At2g28640
-II-	EXO70H6	At1g07725
-II-	EXO70H7	At5g59730
-II-	EXO70H8	At2g28650
Sec3/SEC3	SEC3a	At1g47550
-II-	SEC3b	At1g47560
Sec5/SEC5	SEC5a	At1g76850
-II-	SEC5b	At1g21170
Sec6/SEC6	SEC6	At1g71820
Sec8/SEC8	SEC8	At3g10380
Sec10/SEC10	SEC10a	At5g12370
-II-	SEC10b	NA
Sec15/SEC15	SEC15a	At3g56640
-II-	SEC15b	At4g02350
Exo84/EXO84	EXO84a	At1g10385
-II-	EXO84b	At5g49830
-II-	EXO84c	At1g10180

We will focus here on the most important aspects and news especially related to the plant-specific EXO70 family of subunits and their specificity. The multiplicity of EXO70 paralogs in land plants is impossible to explain exclusively by cell-specific expression, as published transcriptomic data clearly indicate that in every Arabidopsis cell type several EXO70 paralogs are expressed concomitantly. Therefore, different EXO70-specific exocyst complexes might co-exist in the same cell (Zárský et al., 2009, 2013). This is why a hypothesis of specific cortical targeting/recycling domains within a single cell based on specific EXO70s was proposed (Zárský et al., 2009). It is however, also possible that some EXO70 paralogs function outside—i.e., independently of the rest of the exocyst complex (Zhao P. et al., 2013; Zárský et al., 2013).

The first functionally characterized paralog in Arabidopsis was the EXO70A1 subunit. This subunit is abundant and is highly related to other eukaryotic EXO70s (Synek et al., 2006). Despite the expected functional redundancy between the 23 Arabidopsis paralogs (Synek et al., 2006; Cvrčková et al., 2012), single LOF *exo70A1* mutants show a severe phenotype. These mutants are dwarfed with a loss of apical dominance and disruption of polarized cell expansion within cell types that include root hairs and stigma papillae (Synek et al., 2006). Sterility in these plants is caused by the non-receptivity of the stigmatic papillae surface, possibly due to its disturbance during development and maturation (Synek et al., 2006; Safavian et al., 2014). Recently, participation of the whole exocyst complex in stigma function was proven by an RNAi approach that suppressed all core exocyst subunits specifically at the stigmatic surface (Safavian et al., 2015). This (along with other instances—see Zárský et al., 2013) is a clear demonstration that the conclusion of almost exclusive action of the EXO70A1 paralog in xylem secondary thickening (Li et al., 2013) cannot be substantiated by data from other tissues. On the contrary, the original interpretation of EXO70A1 as the most prevalent subunit of exocyst during many exocytotic processes in Arabidopsis seems to be valid (Synek et al., 2006; Fendrych et al., 2013; Zárský et al., 2013), since EXO70A1 LOF affects also polar auxin transport and PM recycling of PIN1/2, PM recycling of receptor-like protein kinase brassinosteroid- insensitive 1 (BRI1), cell wall biogenesis (Drdova et al., 2013; Kulich et al., 2010) and, most importantly, the presence of other exocyst subunits at the PM (Fendrych et al., 2013).

**Figure 2:**
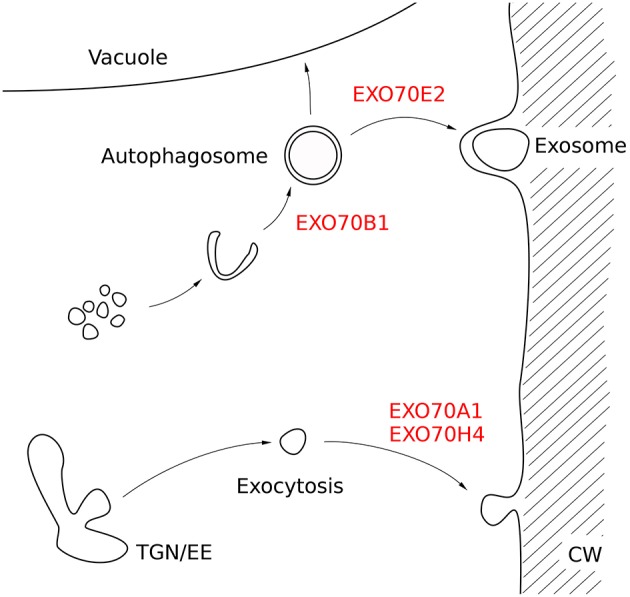
**Modes of action of different EXO70 paralogs in Arabidopsis cell**. Arrows indicate directions of action of EXO70s. EXO70A1 is closely related to other eukaryotic EXO70s that are involved in exocytotic events and it is necessary for the targeting of the core exocyst subunits to plasma membrane, while EXO70H4 is a trichome specific paralog involved in callose secondary cell wall deposition. EXO70B1 regulates autophagy-related membrane transport and EXO70E2 is most probably functioning in both autophagy-related and exosomal pathways.

Unexpectedly, the first clear-cut evidence for EXO70 paralog specificity in plants (Figure 2) came from the discovery that the EXO70B1 subunit is part of a specific version of the exocyst that participates in the regulation of autophagy-related membrane transport pathway from the ER to the vacuole, thus bypassing the GA (Kulich et al., 2013). These data are parallel to the discovery of the exocyst-based platform regulated by Ras-related (RAL) GTPases being involved in the initiation of autophagy in animals (Bodemann et al., 2011). This opens an interesting question whether exocyst engagement in the autophagy in animals and plants is a result of convergent evolution, or possibly an ancient LECA mechanism. Interestingly, EXO70B1 was recently implicated in the regulation of stomatal movement, as *exo70b1* mutants are slow in light induced stomatal opening. The role of EXO70B1 in stomatal opening is mediated by negative regulation via a pathway involving ROP2 and Rac interactive binding motif-containing protein 7 (RIC7), as EXO70B1 function is blocked by its recruitment to the plasma membrane by activated ROP2 mediated by the RIC7 adaptor (Hong et al., 2016). Recently, a specific function of the EXO70H4 subunit in callose secondary cell wall deposition was demonstrated using the model of trichome cell wall maturation (Kulich et al., 2015). Other, even closely related, EXO70 paralogs are unable to complement the LOF phenotypic deviation (Kulich et al., 2015; Kulich et al., in preparation).

Already the extraordinary evolutionary dynamics (Synek et al., 2006; Cvrčková et al., 2012) and transcriptional inducibility by pathogen-associated molecular patterns (PAMPs) and microbe-associated products—elicitors (e.g., elf18, flag22—Pečenková et al., 2011) indicate that some EXO70 paralogs are likely to have a specific roles in the defense against microbial pathogens. T-DNA insertional LOF mutants of EXO70B2 and EXO70H1 paralogs in Arabidopsis are more sensitive to *Pseudomonas syringiae* infection, and the biogenesis of defense papillae against non-adapted fungus *Blumeria graminae* is compromised as well (Pečenková et al., 2011; Stegmann et al., 2012). A specific down-regulation of EXO70B2 protein by degradation mediated by a specific E3 ligase was observed to be part of this biotic interaction (Stegmann et al., 2012), similar to the case of the EXO70A1 subunit and ARC1 E3 ligase in pollen–stigma interactions (Samuel et al., 2009). Therefore, the EXO70-specificity of different plant exocyst complexes might be partially regulated by specific targeted degradation of selected EXO70 paralogs mediated by specific E3 ligases (Zárský et al., 2013). Observed salicylic acid hyperaccumulation in the *exo70B1* mutant points to the participation of the above-discussed EXO70B1 exocyst subunit in Arabidopsis also in defense against microbial pathogens (Kulich et al., 2013; Zhao et al., 2015). Interestingly, a nucleotide binding domain and leucine-rich repeat-containing (NLR)-like disease resistance protein (R protein) TIR-NBS2 (TN2) that lacks the LRR domain is involved in the EXO70B1-mediated biotic interaction. TN2 is proposed to function as a sentinel monitoring EXO70B1 integrity (Zhao et al., 2015). An alternative scenario was proposed recently—that autophagy processes (involving also EXO70B1) might be in some cases involved in the down regulation of plant nucleotide binding domain and leucine-rich repeat-containing (NLR)-like disease resistance (R) proteins (Pečenková et al., 2016). Also, exocyst core subunits are implicated in the warfare between plants and pathogens, since the *Phytophthora infestans* effector avirulence factor 1 (AVR1) specifically targets exocyst subunit SEC5 in potato (Du et al., 2015).

Specificity of putative targeting functions of the EXO70s is based not only on their different molecular structure (see for example the motifs possibly involved in lipid binding or protein stability—Zárský et al., 2009; Cvrčková et al., 2012), but also on the cellular context that may, surprisingly, include interactions with other tethering complexes. In differentiating xylem cells, which deposit highly localized secondary cell wall thickenings, xylem specific coiled-coil proteins VETH1/2 mediate the interaction of EXO70A1 with COG2, recruiting the whole exocyst to the secondary wall deposition domains through interaction with microtubules (Oda et al., 2015; Vukašinović et al., submitted). In contrast, in root epidermal cells of the transition zone, EXO70A1 and the exocyst preferentially localize to the outer/surface cortical PM domain in a microtubule-independent manner (Fendrych et al., 2013). The interaction of some exocyst subunits with COG and TRAPP subunits (see above paragraphs relating to cytokinesis) indicates possible regulatory cross-talk between tethering complexes within the endomembrane system—a feature that will certainly be addressed in the future, not only in plants.

Using ectopic expression of eight Arabidopsis EXO70 paralogs under the 35S promoter in Arabidopsis protoplasts, Wang et al. (2010) observed that individual expression of only three of them—EXO70A1, EXO70B1, and EXO70E2—resulted in the production of a putative new compartment authors called “EXPO” (“exocyst positive organelle”). As both EXO70A1 and EXO70B1 clearly and consistently co-localize with EXO70E2 on the same “EXPOs,” these authors further studied this “organelle” focusing on the EXO70E2 paralog as a true representative of three ″EXPOs″-forming EXO70 paralogs (Wang et al., 2010). Analysing dynamics of these structures they conclude that they do not co-localize with the set of endomembrane markers tested, and that surprisingly these compartments are secreted to the apoplast (Wang et al., 2010). As this behavior would indicate a classic exosomal pathway, the authors also checked autophagy induction and autophagosome markers and concluded that autophagy or multivesicular bodies are either not at all involved in “EXPO” biogenesis and dynamics (Wang et al., 2010), or, that their role may be at most indirect (Lin et al., 2015). However, a detailed analysis of *exo70B1* LOF mutants by Kulich et al. (2013) demonstrated that one of the putative “EXPO” forming EXO70s—EXO70B1—is clearly involved in the autophagy-related processing of imported vacuolar membrane vesicles that bypass the GA (with EXO70B1 co-localizing convincingly with the ATG8 marker). Not only did the LOF mutant produce distinctly more paramural bodies/exosomes, but also overexpression of EXO84b under the 35S promoter produced “EXPOs” while its expression under the natural EXO84b promoter did not (Kulich et al., 2013). Mere ectopic expression of EXO70E2 in animal cells induces “EXPO” formation, indicating that this is an induced artifactual compartment—most probably functioning both as an exosomal or autophagy compartment depending on the cellular context (Ding et al., 2014; Lin et al., 2015). Unfortunately, evidence for EXO70E2 function based on the genetic analysis in Arabidopsis is also missing, so at this point in time “EXPO” compartments cannot be considered as real *in planta/in situ* entities. Circumstantial evidence is currently more compatible with the presence of an autophagy-related and classic exosomal pathway (Zárský et al., 2013).

An important, yet mostly unresolved aspect of exocyst function in plants is the question of if and how RAB and RHO/ROP GTPases might regulate exocyst function. Currently there are at least two known exocyst-GTPase interactions in plants mediated by adaptor proteins. This contrasts with the direct interactions between the exocyst and RHO related GTPases within Opistokonts (Heider and Munson, 2012; Wu and Guo, 2015). In the case of the plant SEC3-ROP interaction the interface protein is interactor of constitutive active ROPs 1 (ICR1) (Lavy et al., 2007) and in the case of EXO70B1 it is RIC7, bridging the interaction with ROP2 in stomata (Hong et al., 2016; see also above). There are no published data available showing the expected exocyst-RAB interactions - it remains to be established if the relevant RAB11/RAB-A paralogs are involved (For many other details of exocyst complex function in plants see Martin-Urdiroz et al., 2016 in this Frontiers Speciality section).

## GARP and EARP complexes

The GARP complex, first described in yeast as a three-subunit complex consisting of vacuolar protein sorting 52 (Vps52p), Vps53p, and Vps54p, is involved in the retrograde transport of Golgi membrane proteins from endosomes and prevacuolar compartments (Conibear and Stevens, 2000; Bonifacino and Hierro, 2011). Additionally the fourth subunit of the complex—Vps51p—was described and found to mediate an interaction between Vps52/53/54 and the t-SNARE Tlg1p (Conibear et al., 2003; Reggiori et al., 2003). The GARP complex is conserved in all eukaryotes (Koumandou et al., 2007). Vps52p, Vps53p, and Vps54p homologs are present as single-copy genes in Arabidopsis (Latijnhouwers et al., 2005), while Vps51p has one homolog with the well-conserved VPS51 domain at its N-terminus and two more homologs with a considerably less conserved N-terminal motif (Pahari et al., 2014; see Table 5).

**Table 5:** **GARP and EARP subunits from yeast and human and their possible homologs in ***Arabidopsis thaliana*****.

	**Yeast/Human subunit**	**Plant subunit**	**Potential *A. thaliana* homolog**
GARP	Vps51/ANG2	VPS51/UNH	At4g02030
	Vps51/ANG2	NA	At1g10385
	Vps51/ANG2	NA	At5g16300
	Vps52/VPS52	VPS52/POK	At1g71300
	Vps53/VPS53	VPS53/HIT1	At1g50500
	Vps54/VPS54	VPS54	At4g19490
EARP	−/Syndetin	NA	At2g27900

In a forward genetic screen a *heat intolerant* (*hit1*) Arabidopsis mutant was isolated (Wu et al., 2000). Follow-up studies showed that the HIT1 gene product is homologous to the yeast Vps53p protein and that it is even able to partially complement heat dependent growth retardation of the yeast vps53Δ mutant (Lee et al., 2006). VPS53/HIT1 localizes to punctae in Arabidopsis protoplasts where it physically interacts with two other putative GARP complex subunits—VPS52/POKY POLLEN TUBE (POK) and VPS54 (Wang et al., 2011), supporting the idea of a functional GARP complex in plants. Moreover, Arabidopsis LOF mutations in *VPS52/POK, VPS53/HIT1*, and *VPS54* genes are all embryonic lethal, suggesting an essential role of this complex during plant development (Lobstein et al., 2004; Guermonprez et al., 2008). These mutants also produce very short pollen tubes with male-specific transmission defects (Lobstein et al., 2004; Guermonprez et al., 2008). This is expected given the presumed vesicle tethering role of the GARP complex in the membrane turnover at the growing pollen tube cell apex. VPS51/UNHINGED (UNH) protein was also shown to be a member of the complex in plants through its interaction with VPS52; surprisingly LOF Arabidopsis mutant plants for this subunit are viable (Pahari et al., 2014). However, these mutants show severe leaf shape and vein patterning developmental defects as a consequence of an impaired targeting of the auxin efflux carrier PIN1 to the lytic vacuole (Pahari et al., 2014). Immunolocalization studies revealed that VPS52/POK co-localizes both with GA and prevacuolar compartment (PVC) markers in sporophyte and gametophyte cells in different plant species (Guermonprez et al., 2008). A recent study confirmed these findings, showing that VPS51/UNH also colocalizes with PVC markers ARA7 and RHAI and Golgi marker SYP61 (Pahari et al., 2014). In yeast, Ypt6p Rab GTPase in its active form recruits VFT complex (Vps52, Vps53, and Vps54) to the late Golgi membranes, thus allowing vesicle fusion (Siniossoglou and Pelham, 2001). A plant homolog of a yeast Ypt6p, RAB-H1b was shown to interact with VPS52 (Osterrieder et al., 2009). This is further support for a supposed conserved function of the GARP complex in plants.

Schindler et al. (2015) recently characterized a human protein syndetin, able to form a novel protein complex EARP, through its interactions with Vps51, Vps52, and Vps53, and showed that this complex (together with a small contribution of GARP), can promote recycling of an internalized cargo to the plasma membrane. BLAST analysis shows that the Arabidopsis At2g27900 locus encodes a protein that shares 29% identity and 44% similarity with syndetin. Whether this putative protein shares the same function as syndetin in plants should be a subject for future research.

## HOPS

Both the HOPS and the CORVET complexes discussed below are heterohexamers. They share four core subunits Vps11, Vps16, Vps18, and Vps33, while two more are specific for each complex (Vps3 and Vps8 for CORVET and Vps39 and Vps41 for HOPS; see Balderhaar and Ungermann, 2013). Yeast Rab7/Ypt7p, located on the vacuole and late endosomes, is activated by the RAB7 GEF complex Mon1–Ccz1 and binds HOPS to enable membrane tethering during homotypic vacuolar fusion (Nordmann et al., 2010).

The Arabidopsis *vacuoleless 1* (*vcl1*) mutant was identified in a screen for mutants with defective early embryo development (Rojo et al., 2001). Cells of this mutant are unable to form vacuoles, have defects in cell elongation and orientation of the cell division plane, and produce large numbers of autophagosomes; all these defects result in embryo lethality at the late torpedo stage. VCL1 is a homolog of yeast Vps16p and is involved in vacuole biogenesis by regulating membrane fusion at the tonoplast (Rojo et al., 2001; see Table 6). In Arabidopsis cells VPS16/VCL1 interacts with two other core HOPS subunits VPS11 and VPS33 and they all localize to the tonoplast and to the prevacuolar compartments (Rojo et al., 2003). Also, SYP2-type syntaxins residing on the prevacuolar compartment, were co-immunoprecipitated using the VPS16/VCL1 directed antibody (Rojo et al., 2003). In the nitrogen-fixing cells of root nodules of *Medicago truncatula*, the expression of both VPS11 and VPS39 HOPS subunits is temporarily switched off at the transition from the infection to the fixation zone of the nodule (Gavrin et al., 2014). Interestingly, during this switch tonoplast-specific integral membrane proteins, such as tonoplast aquaporin TIP1g, are all re-targeted toward symbiosomes in a process essential for the proper functional maturation of the peribacteroid membrane.

**Table 6:** **HOPS and CORVET subunits from yeast and human and their possible homologs in ***Arabidopsis thaliana*****.

	**Yeast/Human subunit**	**Plant subunit**	**Potential *A. thaliana* homolog**
HOPS	Vps11/VPS11	VPS11	At2g05170
	Vps16/VPS16	VPS16/VCL1	At2g38020
	Vps18/VPS18	VPS18	At1g12470
	Vps33/VPS33	VPS33	At3g54860
	Vps39/VPS39	VPS39/EMB2754	At4g36630
	Vps41/VPS41	VPS41/VAM2	At1g08190
CORVET	Vps3/TGFBRAP1	VPS3	At1g22860
	Vps8/VPS8	VPS8	At4g00800

## CORVET

In yeast, the CORVET complex is an effector of the small GTPase Rab5 and functions in endosome–endosome fusion. During the transition to the late endosome, Rab5 is replaced by Rab7, which interacts with HOPS and promotes fusion with the vacuole (Balderhaar and Ungermann, 2013). The presence of a putative complete CORVET complex in plants is predicted by a large phylogenomic comparison. All four common subunits that are shared with the HOPS complex (Vps11, Vps16, Vps18, and Vps33 see above) and two specific ones - Vps3 and Vps8 - are predicted from the Arabidopsis genome (Klinger et al., 2013; Table 6). The only experimental data available are related to LOF mutants of common subunits shared with the HOPS complex, which does not allow any specific functional insights into the function of the CORVET complex in Arabidopsis. This implies, at the same time, that some phenotypic deviations described in the HOPS subunit mutant plants described above, might be due to perturbation of the CORVET complex. RAB5 GTPases are potential regulators of this complex and have some plant-specific features. It can therefore be expected that the HOPS/CORVET complexes will also have plant-specific features (see the Introduction).

## Conclusions

Tethering complexes are undoubtedly key players in the membrane trafficking events also in plant cells. Even with the limited experimental data currently available, it is apparent that the endomembrane vesicle tethering complexes are essential for numerous growth and developmental processes in plants. Often, mutations in single/“orphan” genes coding for the core subunits of tethering complexes are gametophytic or embryonic lethal. However, in many cases subunits of these complexes are encoded by several paralogs. In the case of the EXO70 subunit of the land plant exocyst complex, tens of paralogs did evolve within a single species, indicating a functional diversification of the terminal phase of the secretory pathway in plants. Such diversification of exocytosis in different cell types and specific cell cortical domains is a feature fully compatible with the sessile life style of cell wall-possessing plants. Most of the work in the characterization of tethering mechanisms within the plant cell still lay ahead. Approaches that will include simultaneous, tissue specific and inducible knockdowns of genes involved will shed new light on the function and regulation of tethering complexes and their interacting partners in plants. As indicated (for example) by the observations discussed previously concerning the Dsl1 complex, forward screens and proteomic analyses will inevitably identify new, plant specific subunits of tethering complexes and possibly even entirely novel complexes in the future. The control of the tethering complexes by activated GTPases, as well as regulation of GTPases such as RAB by the tethering complexes themselves, is a crucial aspect of membrane trafficking logistics in plant cells that urgently needs more attention. Hints that indicate possible functional interactions of different tethering complexes that have appeared recently (e.g., exocyst-COG; exocyst-TRAPP see above) might imply that we have to expect an extensive cross-talk and coordination between different endomembrane tethering complexes in eukaryotic cells in general. Since secretory pathways are at the heart of plant growth and development, a better theoretical understanding of their regulation will inevitably provide powerful benefits for agriculture.

## Author contributions

All authors listed, have made substantial, direct and intellectual contribution to the work, and approved it for publication.

### Conflict of interest statement

The authors declare that the research was conducted in the absence of any commercial or financial relationships that could be construed as a potential conflict of interest.
